# Copper(I)-Catalyzed [ 3+ 2] Cycloaddition of 3-Azidoquinoline-2,4(1*H*,3*H*)-diones with Terminal Alkynes ^†^

**DOI:** 10.3390/molecules16054070

**Published:** 2011-05-18

**Authors:** Stanislav Kafka, Sylvia Hauke, Arjana Salcinovic, Otto Soidinsalo, Damijana Urankar, Janez Kosmrlj

**Affiliations:** 1 Department of Chemistry, Faculty of Technology, Tomas Bata University in Zlin, Zlin 76272, Czech Republic; 2 Institute of Chemistry, University of Potsdam, Golm D-14476, Germany; 3 Faculty of Natural Sciences and Mathematics, University of Banja Luka, Banja Luka 78000, Bosnia and Herzegovina; 4 Department of Chemistry, Faculty of Science, University of Helsinki, Helsinki FI-00014, Finland; 5 Faculty of Chemistry and Chemical Technology, University of Ljubljana, Ljubljana SI-1000, Slovenia

**Keywords:** cycloaddition, azides, quinoline-2,4(1*H*,3*H*)-diones, terminal alkynes, 1,2,3-triazoles

## Abstract

3-Azidoquinoline-2,4(1*H*,3*H*)-diones **1**, which are readily available from 4-hydroxyquinolin-2(1*H*)-ones **4** *via* 3-chloroquinoline-2,4(1*H*,3*H*)-diones **5**, afford, in copper(I)-catalyzed [3 + 2] cycloaddition reaction with terminal acetylenes, 1,4-disubstituted 1,2,3-triazoles **3** in moderate to excellent yields. The structures of compounds **3** were confirmed by ^1^H and ^13^C-NMR spectroscopy, combustion analyses and mass spectrometry.

## 1. Introduction

**Scheme 1:**
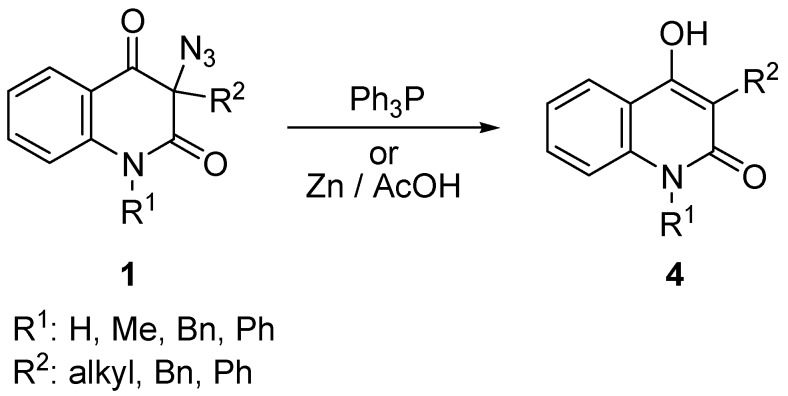
Previously documented unexpected reactivity of 3-azidoquinoline-2,4(1*H*,3*H*)-diones **1**.

As a part of our continuous interest in quinolinedione chemistry, we observed that 3-azidoquinoline-2,4(1*H*,3*H*)-diones **1** behave quite differently than normally expected for organic azides, including α-azido carbonyl compounds [1]. For example, the Staudinger reaction [2], the reduction of azide with triphenylphosphine, did not yield the expected 3-aminoquinoline-2,4(1*H*,3*H*)-diones (Scheme 1) and instead, deazidation took place to afford 4-hydroxyquinolin-2(1*H*)-ones. Similar behaviour of 3-azidoquinoline-2,4(1*H*,3*H*)-diones was also observed in the reaction with zinc in acetic acid [1].

This unexpected reactivity prompted us to continue the studies of 3-azidoquinoline-2,4(1*H*,3*H*)-diones reactivity. For examples, we were intrigued whether these compounds could serve as partners in copper(I)-catalyzed [3 + 2] cycloaddition reaction with terminal alkynes. This reaction, also referred to as “Click reaction”, has been recently discovered to selectively afford 1,4-disubstituted 1,2,3-triazoles [3,4,5]. Its remarkably mild reaction conditions, broad scope, and exquisite selectivity are well documented, and it has succeeded in the presence of most functional groups tested to date. It has also become a powerful and versatile tool in nearly all areas of chemistry, including macromolecular engineering, nanotechnology, and drug discovery [6,7,8,9,10].

## 2. Results and Discussion

Starting 3-azidoquinoline-2,4(1*H*,3*H*)-diones **1** were prepared by known chemistry from the corresponding 4-hydroxyquinolin-2-(1*H*)-ones **4**. Chlorination and bromination of the latter with sulfuryl chloride and bromine, respectively, afforded the corresponding 3-chloroquinoline-2,4(1*H*,3*H*)-diones **5** and 3-bromoquinoline-2,4(1*H*,3*H*)-diones **6**. The resulting 3-halogenoquinoline-2,4(1*H*,3*H*)-diones were subsequently subjected to the substitution with sodium azide.

**Scheme 2:**
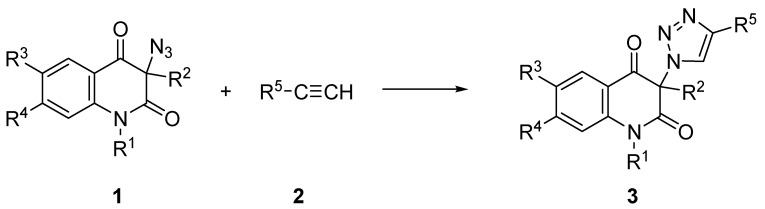
Copper(I) catalyzed cycloaddition between 3-azidoquinoline-2,4(1*H*,3*H*)-diones **1** and terminal acetylenes into 1,2,3-triazoles **3**.

3-Azidoquinoline-2,4(1*H*,3*H*)-diones **1** were examined as partners in copper(I) catalyzed [3 + 2] cycloaddition (Scheme 2). Three different terminal acetylenes **2** were chosen; phenylacetylene (**2a**), propargyl alcohol (**2b**) and 3-ethynylaniline (**2c**). When screening for the optimal reaction conditions, we initially tested the most commonly used system, copper(II) sulphate pentahydrate and ascorbic acid as a source of copper(I) in *tert*-BuOH/H_2_O as a solvent [6]. Interestingly, no reaction could be detected by thin-layer chromatography (TLC) analysis after 24 h and the starting azides **1** were recovered nearly quantitatively from the reaction mixtures. Similarly unsuccessful were attempts to use a combination of copper(II) acetate and elemental copper in acetonitrile. We assumed that the prime reasons for the failure of these reactions were the extremely low solubilities of azides **1** in the reaction media used. Similar difficulties were previously encountered by some of us in attempts at using sparingly soluble propargyl functionalized diazenecarboxamides [11] or azido-appended platinum(II) complexes [12] as click components. In those instances the use of dimethyl sulfoxide (DMSO) as a reaction solvent and a combination of copper(II) sulphate pentahydrate and elemental copper (CuSO_4_/Cu^(0)^) provided results that were superior to other combinations. Conducting the cycloadditions between azides **1** and acetylenes **2** in DMSO, in the presence of CuSO_4_/Cu^(0)^ couple afforded the expected 1,4-disubstituted 1,2,3-triazoles **3** in moderate to excellent yields, as shown in Table 1.

**Table 1:** Reaction conditions and yields for the formation of 1,2,3-triazoles **3** (Scheme 2) ^a^.

**Entry**	**Azide**					**Alkyne**		**Triazole**	**R. time, h**	**Yield, %^ b^**
	**1**	R^1^	R^2^	R^3^	R^4^	**2**	R^5^	**3**		
**1**	**1A**	H	Ph	H	H	**2a**	Ph	**3Aa**	3.3	96 (80)
**2**	**1A**	H	Ph	H	H	**2b**	CH_2_OH	**3Ab**	1.5	95 (67)
**3**	**1A**	H	Ph	H	H	**2c**	3-(NH_2_)-C_6_H_4_	**3Ac**	1.3	nd (61)
**4**	**1B**	H	Ph	MeO	H	**2a**	Ph	**3Ba**	2.2	98 (64)
**5**	**1B**	H	Ph	MeO	H	**2b**	CH_2_OH	**3Bb**	1.1	85 (nd)
**6**	**1C**	H	Pr	MeO	H	**2a**	Ph	**3Ca**	1.2	97 (78)
**7**	**1C**	H	Pr	MeO	H	**2b**	CH_2_OH	**3Cb**	3	nd (60)
**8**	**1D**	H	Ph	Cl	MeO	**2a**	Ph	**3Da**	1.2	92 (82)
**9**	**1D**	H	Ph	Cl	MeO	**2b**	CH_2_OH	**3Db**	3	nd (46)
**10**	**1E**	Bn	Ph	H	H	**2a**	Ph	**3Ea**	2.5	97 (51)
**11**	**1E**	Bn	Ph	H	H	**2b**	CH_2_OH	**3Eb**	2	94 (89)

^a^ Reaction conditions: **1** (2.00 mmol), **2** (2.02 mmol), CuSO_4_∙5H_2_O (0.2 mmol, 10 mol %), granular copper (8.8 mmol), DMSO (6 mL), air, room temperature, darkness; ^b^ Chemical yield of NMR pure, crude isolated product. The value in parentheses refers to the yield of crystallized product; nd: not determined.

In a general procedure, a mixture of 3-azidoquinoline-2,4(1*H*,3*H*)-dione (**1**, 2.0 mmol), terminal alkyne (**2**, 2.0 mmol), CuSO_4_∙5H_2_O (0.2 mmol, 10 mol %), and granular copper (8.8 mmol) in DMSO (6 mL) was stirred at room temperature, in the presence of air. Since azides **1** are prone to slow decomposition in the presence of light, we decided to perform the reactions in darkness. The cycloadditions were completed within a few hours (Table 1), and as judged by TLC analyses, the corresponding 1,4-disubstituted 1,2,3-triazoles **3** were formed quantitatively. In most cases the products were isolated by simple extractive workup in excellent chemical yields and more than 95% purity as judged by ^1^H-NMR and TLC analyses (Table 1, entries 1, 2, 4–6, 8, 10 and 11). The relatively high loads of granular copper could easily be recovered and eventually reused.

**Figure 1:**
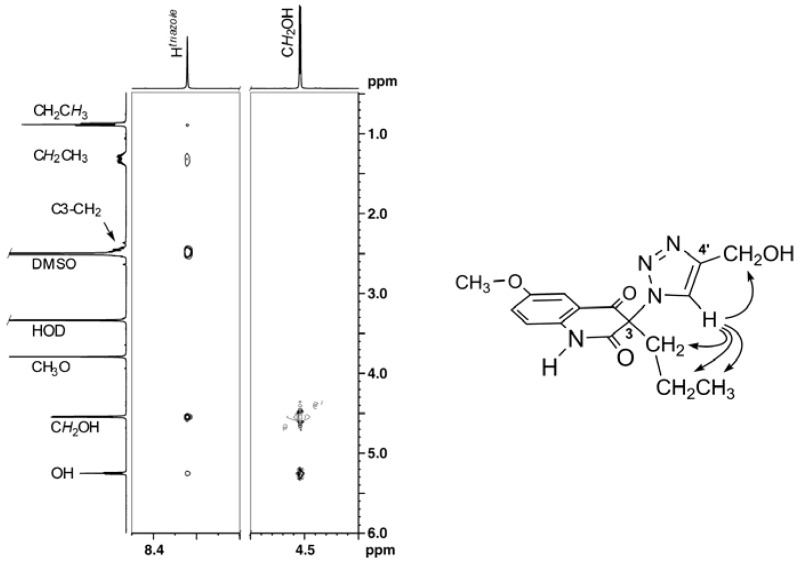
Expansions of NOESY spectrum and chemical drawing of compound **3Cb** showing relevant NOE cross peaks.

The structures of triazoles **3** were confirmed by ^1^H- and ^13^C-NMR spectroscopy, combustion analyses and mass spectrometry on the crystallized compounds. In one instance, that of **3Cb**, the expected 1,4-regiochemistry at the 1,2,3-triazole ring was confirmed by a NOESY experiment. As demonstrated in Figure 1, the triazole hydrogen atom (H^triazole^) displays five NOE cross peaks; three to the propyl group bound to C3 of the quinolinedione core and two to the hydroxymethyl group, attached to C4’ of the triazole ring. The most important for the assigned regiochemistry is the cross peak of H^triazole^ to the C3-CH_2_ protons, which would not be possible for the isomeric 1,5-disubstituted product. The absence of a cross peak between the hydroxymethyl group and C3-CH_2_ further corroborates the structure of **3Cb**.

In one instance, that of 3-phenyl-3-(4-phenyl-1*H*-1,2,3-triazol-1-yl)quinoline-2,4(1*H*,3*H*)-dione(**3Aa**), the preparation of 3-azido-3-phenylsquinoline-2,4(1*H*,3*H*)-dione (**1A**) and its cycloaddition with phenylacetylene were conducted as a one-pot multicomponent reaction, *i.e.*, by mixing the corresponding 3-bromoquinolinedione substrate **6A**, sodium azide and alkyne (**2a**) in the presence of Cu^(II)^/Cu^(0)^ (Experimental section). Similar one-pot azidation-cycloaddition procedures are described in the literature [13,14] This protocol afforded the desired product (**3Aa**) in modest 43% yield. 3-(4-(3-Aminophenyl)-1*H*-1,2,3-triazol-1-yl)-3-phenylquinoline-2,4(1*H*,3*H*)-dione (**3Ac**) was acetylated into *N*-(3-(1-(1,2,3,4-tetrahydro-2,4-dioxo-3-phenylquinolin-3-yl)-1*H*-1,2,3-triazol-4-yl)phenyl)-acetamide (**3Ae**). Whereas α-azido-β-carbonyl lactam has previously been used as a reaction partner [15], to the best our knowledge, this is not the case with 3-azidoquinoline-2,4(1*H*,3*H*)-diones.

## 3. Experimental

### 3.1. General

**Scheme 3:**
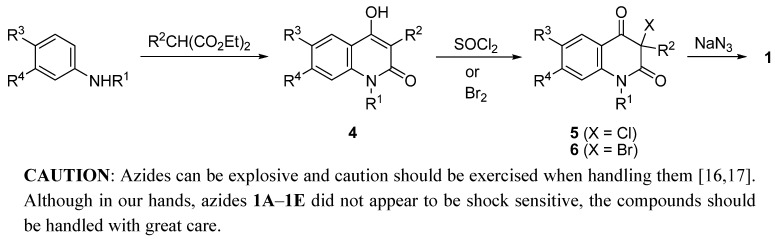
Preparation of starting compounds **4**–**6**. For key to substituents, please see Table 1.

Reagents and solvents were commercially sourced (Fluka, Aldrich, Alfa Aesar) and used as purchased. Granular copper (particle size 0.2–0.7 mm), coating quality (99.9%, Fluka #61144) was used. For column chromatography, Fluka Silica gel 60, 220–440 mesh was used. The course of separation and also the purity of substances were monitored by TLC on Alugram® SIL G/UV254 foils (Macherey-Nagel). NMR spectra were recorded at 302 K on a Bruker Avance DPX 300 spectrometer operating at 300 MHz (^1^H) and 75 MHz (^13^C), and Bruker Avance III 500 MHz NMR instrument operating at 500 MHz (^1^H) and 125 MHz (^13^C). Proton spectra were referenced to TMS as internal standard. Carbon chemical shifts were determined relative to the ^13^C signal of DMSO-*d*_6_ (39.5 ppm). Chemical shifts are given on the δ scale (ppm). Coupling constants (*J*) are given in Hz. Multiplicities are indicated as follows: s (singlet), d (doublet), t (triplet), q (quartet), m (multiplet), or br (broadened). Phase sensitive NOESY with gradient pulses in mixing time, of **3Cb**, was recorded in DMSO-*d*_6_(c = 21 mM) using standard pulse sequence from the Bruker pulse library (noesygpphpp in the Bruker software) at 296 K, with mixing time of 300 ms and relaxation delay of 2 s. Mass spectra and high-resolution mass spectra were obtained with a VG-Analytical AutospecQ instrument and Q-TOF Premier instrument. Data are reported as *m/z* (relative intensity). The IR spectra were recorded on a Perkin-Elmer 421 and 1310 and Mattson 3000 spectrophotometers using samples in potassium bromide disks. Elemental analyses (C, H, N) were performed with FlashEA1112 Automatic Elemental Analyzer (Thermo Fisher Scientific Inc.). The melting points were determined on a Kofler block or Gallenkamp apparatus and are uncorrected. Starting compounds **1**, **4–6** were prepared by known procedures as shown in Scheme 3 and described below.

4-Hydroxyquinolin-2-(1*H*)-ones: 4-Hydroxy-3-phenylquinolin-2(1*H*)-one (**4A**) [18], 4-hydroxy-6-methoxy-3-phenylquinolin-2(1*H*)-one (**4B**) [19], 6-chloro-4-hydroxy-7-methoxy-3-phenylquinolin-2(1*H*)-one (**4D**) [19], and 1-benzyl-4-hydroxy-3-phenylquinolin-2(1*H*)-one (**4E**) [1] were prepared by thermal condensation of the appropriate anilines and substituted malonic acids diethyl esters neat, as described in the literature. 1-Benzyl-3-chloro-3-phenylquinoline-2,4(1*H*,3*H*)-dione (**5E**) was prepared by chlorination of compound **4E** with sulfuryl chloride according to the literature procedure [1]. 3-Bromo-3-phenylquinoline-2,4(1*H*,3*H*)-dione (**6A**) and 3-bromo-6-methoxy-3-phenylquinoline-2,4-(1*H*,3*H*)-dione (**6B**) were prepared by bromination of compounds **4A** and **4B**, respectively, as described in the literature [20]. 3-Azido-1-benzyl-3-phenylquinoline-2,4(1*H*,3*H*)-dione (**1E**) was prepared by the reaction of 1-benzyl-3-chloro-3-phenylquinoline-2,4(1*H*,3*H*)-dione (**5E**) with sodium azide as described formerly [1].

### 3.2. 4-Hydroxy-6-methoxy-3-propylquinolin-2(1H)-one ***(4C)***

A mixture of 4-methoxyaniline (6.16 g, 50 mmol) and diethyl propylmalonate (10.52 g, 52 mmol) was heated on a metal bath at 220–230 °C for 1 h and then at 260–280 °C for 3 h (the reaction was complete when the distillation of ethanol stopped). After cooling, the solid product was crushed, suspended in aqueous sodium hydroxide solution (0.5 M, 125 mL) and after filtration the filtrate was washed with toluene (3 × 20 mL). The aqueous phase was filtered and acidified by concentrated hydrochloric acid. The precipitated crude product was filtered, washed with water, air dried and crystallized from ethanol affording white solid of **4C**, yield 5.90 g (51%); mp 238–243 °C; IR (cm^−1^): 2961, 1644 (CO), 1618, 1607, 1591, 1453, 1272, 1240, 1202, 1164; ^1^H-NMR (DMSO-*d*_6_) 0.91 (t, *J* = 7.3 Hz, 3H), 1.39–1.51 (m, 2H), 2.50–2.56 (m, 2H), 3.79 (s, 3H), 7.08 (dd, *J* = 9.0, 2.7 Hz, 1H), 7.19 (d, *J* = 9.1 Hz, 1H), 7.36 (d, *J* = 2.6 Hz, 1H), 9.89 (s, 1H), 11.14 (s, 1H); ^13^C-NMR (DMSO-*d*_6_) 13.9, 21.3, 25.0, 55.4, 104.2, 111.9, 115.7, 116.1, 118.6, 131.8, 153.7, 156.5, 163.0; MS (EI) *m/z* (%): 233 ([M]^+^, 97), 218 (92), 205 (97), 204 (100), 191 (37); Anal. Calcd for C_13_H_15_NO_3_ (233.26): C, 66.94; H, 6.48; N, 6.00; Found: C, 67.07; H, 6.55; N, 6.21.

### 3.3. 3-Chloro-6-methoxy-3-propylquinoline-2,4(1H,3H)-dione ***(5C)***

To a stirred suspension of 4-hydroxy-6-methoxy-3-propylquinolin-2(1*H*)-one (**4C**, 3.97 g, 17 mmol) in dioxane (50 mL) preheated to 50 °C, sulfuryl chloride (7.8 g, 57 mmol) was added dropwise, keeping the temperature below 55 °C. The resulting reaction mixture was stirred for additional 10 min at 50–55 °C, cooled down to room temperature and poured into ice-water (850 mL). The precipitated crude product was filtered, washed with water (200 mL), air dried and crystallized from benzene affording bright yellow solid of **5C**, yield 3.48 g (76%); mp 138–142 °C; IR (cm^−1^): 2918, 1703 (CO), 1672 (CO), 1504, 1430, 1288, 1203; ^1^H-NMR (DMSO-*d*_6_) 0.86 (t, *J* = 7.2 Hz), 1.12–1.30 (m, 2H), 2.20–2.29 (m, 2H), 3.80 (s, 3H), 7.11 (d, *J* = 8.7 Hz, 1H), 7.30 (dd, *J* = 7.2, 3.0 Hz, 1H), 7.34 (d, *J* = 3.0 Hz, 1H), 11.16 (s, 1H); ^13^C-NMR (DMSO-*d*_6_) 13.8, 17.9, 37.9, 55.6, 66.7, 109.0, 117.6, 118.3, 125.3, 135.1, 155.1, 166.3, 188.4; MS (EI) *m/z* (%): 269 ([M{^37^Cl}]^+^, 17), 267 ([M{^35^Cl}]^+^, 42), 240 (48), 238 (100), 233 (33), 225 (52); Anal. Calcd for C_13_H_14_ClNO_3_ (267.71): C, 58.32; H, 5.27; N, 5.23. Found: C, 58.58; H, 5.07; N, 5.36.

### 3.4. Chlorination of 6-chloro-4-hydroxy-7-methoxy-3-phenylquinolin-2(1H)-one ***(4D)*** into 3,6-Dichloro-7-methoxy-3-phenylquinoline-2,4(1H,3H)-dione ***(5D)*** and 3,6,8-Trichloro-7-methoxy-3-phenylquinoline-2,4(1H,3H)-dione ***(5F)***

Sulfuryl chloride (16.7 g; 124 mmol) was added dropwise to the stirred suspension of 6-chloro-4-hydroxy-7-methoxy-3-phenylquinolin-2(1*H*)-one (**4D**, 10.00 g; 37.41 mmol) in dioxane at 46–47 °C during 20 min. The resulting reaction mixture was stirred for 10 min and poured into ice-water (600 mL). The precipitated solid was filtered, washed with water, dried on the air and crystallized from benzene (1.3 L) affording compound **5D**. The mother liquor was concentrated *in vacuo* to approximately 380 mL. The precipitated solid was filtered and recrystallized from benzene to give compound **5F**.

*3,6-Dichloro-7-methoxy-3-phenylquinoline-2,4(1H,3H)-dione* (**5D**): White solid, yield 6.33 g (50%); mp 236–238 °C (236 °C from acetic acid [20]); IR (cm^−1^): 1710 (CO), 1679 (CO), 1614, 1595, 1485, 1445, 1400, 1335; ^1^H-NMR (DMSO-*d_6_*) 3.93 (s, 3H, CH_3_), 6.84 (s, 1H), 7.29–7.38 (m, 5H), 7.66 (s, 1H), 11.13 (s, 1H); ^13^C-NMR (DMSO-*d_6_*) 56.8, 74.4, 100.0, 111.4, 116.7, 127.0, 128.5, 128.8, 129.3, 134.9, 141.9, 160.6, 166.6, 185.5; MS (EI) *m/z* (%): 339 ([M{^37^Cl_2_}]^+^, 6), 337 ([M{^37^Cl^35^Cl}]^+^, 31), 335 ([M{^35^Cl_2_}]^+^, 42), 303 (45), 302 (54), 301 (100), 300 (83), 186 (28), 184 (67), 183 (32); Anal. Calcd for C_16_H_11_Cl_2_NO_3_ (336,17): C, 57.16; H, 3.30; N, 4.17. Found: C, 57.16; H, 3.26; N, 4.10.

*3,6,8-Trichloro-7-methoxy-3-phenylquinoline-2,4(1H,3H)-dione* (**5F**): White solid, yield 0.82 g (6%); mp 183–186 °C (198 °C from pet. ether [20]); IR (cm^−1^): 3238 (NH), 1728 (CO), 1696 (CO), 1596, 1463, 1317, 1055, 966, 749, 692; MS (EI) *m/z* (%): 373 ([M{^37^Cl_2_^35^Cl}]^+^, 4), 371 ([M{^37^Cl^35^Cl_2_}]^+^, 11), 369 ([M{^35^Cl_3_}]^+^, 11), 338 (20), 337 (63), 336 (78), 335 (99), 334 (100), 220 (27), 218 (41), 89 (27). Anal. Calcd for C_16_H_10_Cl_3_NO_3_ (370.61): C, 51.85; H, 2.72; N, 3.78. Found: C, 51.92; H, 2.70; N, 3.61.

### 3.5. General Procedure for the Synthesis 3-Azidoquinoline-2,4(1H,3H)-diones ***1A–D***

To a stirred solution of the appropriate 3-chloro- (**5A**,**B**) or 3-bromoquinoline-2,4(1*H*,3*H*)-dione (**6C**,**D**, 10 mmol) in DMF (50 mL), sodium azide (975 mg, 15 mmol) was added in small portions in darkness during 20 min. The reaction mixture was stirred in darkness for 2 h and then poured into ice-water (600 mL). The precipitated product **1** was filtered, washed with water (150 mL) and dried at 50–60 °C in darkness. Products **1A**, **1C**, **1D** were crystallized from the solvents indicated below.

*3-Azido-3-phenylquinoline-2,4(1H,3H)-dione* (**1A**): White solid, yield 2.13 g (77%); mp 173–181 °C (benzene; 170–172 °C [1]).

*3-Azido-6-methoxy-3-phenyquinoline-2,4(1H,3H)-dione* (**1B**): Yellow solid, yield 2.76 g (90%); mp 183–185 °C (182–183 °C from ethanol [20]). Anal. Calcd for C_16_H_12_N_4_O_3_ (308.29): C, 62.33; H, 3.92; N, 18.17. Found: C, 62.21; H, 4.09; N, 18.22.

*3-Azido-6-methoxy-3-propylquinoline-2,4(1H,3H)-dione* (**1C**): Yellow solid, yield 2.39 g (87%); mp 170–171 °C (ethanol); IR (cm^−1^): 2962, 2121 (N_3_), 1700 (CO), 1665 (CO), 1509, 1492, 1424, 1354, 1208, 826; ^1^H-NMR (DMSO-*d_6_*) 0.81 (t, *J* = 7.2 Hz, 3H), 1.20–1.32 (m, 2H), 1.80–1.97 (m, 2H), 3.78 (s, 3H), 7.06 (d, *J* = 8.8 Hz, 1H), 7.21 (d, *J* = 3.0 Hz, 1H), 7.28 (dd, *J* = 8.8, 3.0 Hz, 1H), 10.96 (s, 1H); ^1^H-NMR (CDCl_3_) 0.91 (t, *J* = 7.3 Hz, 3H), 1.37–1.49 (m, 2H), 2.04–2.22 (m, 2H), 3.85 (s, 3H), 7.00 (d, *J* = 8.7 Hz, 1H), 7.19 (dd, *J* = 8.9, 2.8 Hz, 1H), 7.38 (d, *J* = 2.8 Hz, 1H), 9.58 (s, 1H); ^13^C-NMR (DMSO-*d_6_*) 13.4, 16.7, 39.2, 55.5, 74.5, 108.7, 118.1, 118.9, 124.5, 135.2, 154.9, 168.5, 191.2; ^13^C-NMR (CDCl_3_) 13.7, 17.1, 39.9, 55.8, 73.6, 109.4, 118.1, 119.3, 125.3, 133.9, 156.4, 170.5, 191.6; MS (EI) *m/z* (%): ([M]^+^, 30), 218 (28), 149 (100), 121 (39), 106 (66); Anal. Calcd for C_13_H_14_N_4_O_3_ (274.28): C, 56.93; H, 5.14; N, 20.43. Found: C, 56.72; H, 5.36; N, 20.37.

*3-Azido-6-chloro-7-methoxy-3-phenylquinoline-2,4(1H,3H)-dione* (**1D**): White solid, yield 3.14 g (92%); mp 228–232 °C dec. (ethanol); IR (cm^−1^): 2976, 2920, 2126 (N_3_), 1710 (CO), 1670 (CO), 1613, 1590, 1407, 1348, 1279; ^1^H-NMR (DMSO-*d_6_*) 3.91 (s, 3H, CH_3_), 6.73 (s, 1H), 7.35–7.41 (m, 2H), 7.41–7.49 (m, 3H), 7.72 (s, 1H), 11.42 (s, 1H); ^13^C-NMR (DMSO-*d_6_*) 56.7, 76.8, 100.0, 112.3, 116.5, 126.6, 128.1, 129.6, 130.0, 133.3, 141.9, 160.3, 168.1, 187.1; MS (EI) *m/z* (%): 344 ([M{^37^Cl}]^+^, 3), 342 ([M{^35^Cl}]^+^, 9), 318 (11), 316 (40), 314 (22), 303 (17), 302 (21), 301 (51), 300 (38), 183 (100); Anal. Calcd for C_16_H_11_ClN_4_O_3_ (342.74): C, 56.07; H, 3.23; N, 16.35. Found: C, 56.30; H, 3.24; N, 16.45.

### 3.6. General Procedure for the Preparation of 1,2,3-Triazoles ***3***

A mixture of 3-azidoquinoline-2,4(1*H*,3*H*)-dione (**1**, 2.00 mmol), terminal alkyne (**2**, 2.02 mmol), CuSO_4_×5H_2_O (0.2 mmol, 10 mol%), granular copper (8.8 mmol), and DMSO (6 mL) was stirred at room temperature in darkness until the starting compound **1** became undetectable by TLC (The reaction times are indicated in Table 1). Then the reaction mixture was diluted with CH_2_Cl_2_ (160–250 mL) and filtered. The filtrate was washed with saturated aqueous NH_4_Cl (3 × 80 mL) until the aqueous layer remained colourless (concentrated aqueous ammonia (0.25 mL) was added to the saturated aqueous NH_4_Cl for the isolation of **3Ac**. Each time the product was back-extracted from the water layer with few millilitres of CH_2_Cl_2_. The combined organic layers were shortly dried over Na_2_SO_4_, filtered, and the solvents were evaporated *in vacuo*. Residual DMSO was removed by several consecutive co-distillations *in vacuo* with toluene and then ethanol. The product was suspended in boiling cyclohexane (20 mL), cooled down to room temperature, filtered and dried to give the corresponding triazole **3**. For analyses the products were crystallized from the solvent indicated below. Reaction times along with the yields of crude and crystallized products are indicated in Table 1.

*3-Phenyl-3-(4-phenyl-1H-1,2,3-triazol-1-yl)quinoline-2,4(1H,3H)-dione* (**3Aa**): White solid; mp 274–277 °C (ethanol); IR (cm^−1^): 3276, 1721 (CO), 1690 (CO), 1613, 1485, 1353, 771, 756; ^1^H-NMR (DMSO-*d_6_*) 7.10–7.21 (m, 2H), 7.29–7.39 (m, 1H), 7.39–7.49 (m, 4H), 7.49–7.57 (m, 3H), 7.60–7.69 (m, 1H), 7.79–7.92 (m, 3H), 8.48 (s, 1H), 11.66 (br s, 1H); ^13^C-NMR (DMSO-*d_6_*) 80.0, 116.7, 119.1, 123.4, 123.5, 125.1, 127.5, 127.9, 128.8, 128.9, 129.5, 129.9, 130.49, 130.52, 136.9, 140.5, 145.3, 166.7, 188.9; HRMS (ESI+) calcd for C_23_H_17_N_4_O_2_ ([M + H]^+^): 381.1357, found 381.1355; Anal. Calcd for C_23_H_16_N_4_O_2_ (380.40): C, 72.62; H, 4.24; N, 14.73; Found: C, 72.59; H, 4.24; N, 14.54.

*3-(4-(Hydroxymethyl)-1H-1,2,3-triazol-1-yl)-3-phenylquinoline-2,4(1H,3H)-dione* (**3Ab**): Yellow-brown solid; mp 116–135 °C (benzene); IR (cm^−1^): 3425, 2915, 1721 (CO), 1684 (CO), 1613, 1595, 1485, 1354, 762; ^1^H-NMR (DMSO-*d_6_*) 4.53 (d, *J* = 5.4 Hz, 2H), 5.20 (br t, *J* = 5.4 Hz, 1H), 7.10 (d, *J* = 8.1 Hz, 1H), 7.16 (dd, *J* = 7.5, 7.5 Hz, 1H), 7.34–7.45 (m, 2H), 7.45–7.56 (m, 3H), 7.62 (dd, *J* = 7.5, 7.5 Hz, 1H), 7.75 (s, 1H), 7.84 (d, *J* = 7.5 Hz, 1H), 11.59 (br s, 1H); ^13^C-NMR (DMSO-*d_6_*) 55.0, 79.6, 116.6, 119.1, 123.4, 124.7, 127.5, 128.7, 129.5, 130.2, 130.5, 136.8, 140.5, 146.8, 166.8, 188.9. Anal. Calcd for C_18_H_14_N_4_O_3_ (334.33): C, 64.66; H, 4.22; N, 16.76; Found: C, 64.86; H, 4.41; N, 16.62. 

*3-(4-(3-Aminophenyl)-1H-1,2,3-triazol-1-yl)-3-phenylquinoline-2,4(1H,3H)-dione* (**3Ac**): Brown solid, mp 273–277 °C (DMF-ethanol); IR (cm^−1^): 3366, 1720 (CO), 1690 (CO); 1613, 1593, 1484, 1353, 774, 757, 697; ^1^H-NMR (DMSO-*d*_6_) 5.16 (br s, 2H), 6.49–6.55 (m, 1H), 6.90 (d, *J* = 7.7 Hz, 1H), 7.01–7.13 (m, 3H), 7.17 (dd, *J* = 7.6, 7.6 Hz, 1H), 7.37–7.46 (m, 2H), 7.47–7.56 (m, 3H), 7.59–7.68 (m, 1H), 7.85 (dd, *J* = 7.8, 1.2 Hz, 1H), 8.27 (s, 1H), 11.59 (br s, 1H); ^13^C-NMR (DMSO-*d*_6_) 79.9, 110.3, 112.8, 113.5, 116.5, 119.1, 122.8, 123.3, 127.4, 128.8, 129.3, 129.4, 129.9, 130.4, 130.9, 136.7, 140.4, 145.9, 149.0, 166.8, 188.9; HRMS (ESI+) calcd for C_23_H_18_N_5_O_2_ ([M + H]^+^): 396.1460, found 396.1475. 

*6-Methoxy-3-phenyl-3-(4-phenyl-1H-1,2,3-triazol-1-yl)quinoline-2,4(1H,3H)-dione* (**3Ba**): Yellow solid; mp 138–145 °C (benzene); IR (cm^−1^): 3077, 1720 (CO), 1681 (CO), 1502, 1419, 1345, 756, 695; ^1^H-NMR (DMSO-d_6_) 3.78 (s, 3H), 7.09 (d, *J* = 8.5 Hz, 1H), 7.23–7.59 (m, 10H), 7.83 (d, *J* = 7.4 Hz, 2H), 8.44 (br s, 1H), 11.51 (br s, 1H); ^13^C-NMR (DMSO-*d_6_*) 55.6, 79.7, 109.3, 118.3, 119.5, 123.3, 124.9, 125.1, 127.9, 128.8, 128.9, 129.5, 130.0, 130.48, 130.51, 134.4, 145.2, 155.2, 166.3, 188.9; MS (EI) *m/z* (%): 411 ([M+1]^+^, 3), 410 ([M]^+^, 13), 277 (16), 267 (42), 266 (77), 251 (18), 223 (12), 145 (13), 117 (13), 116 (100), 106 (15), 102 (14), 89 (21), 77 (15); HRMS (ESI+) calcd for C_24_H_19_N_4_O_3_ ([M + H]^+^): 411.1457, found 411.1444.

*3-(4-(Hydroxymethyl)-1H-1,2,3-triazol-1-yl)-6-methoxy-3-phenylquinoline-2,4(1H,3H)-dione* (**3Bb**): Yellow solid; mp 230–235 °C (crude product); IR (cm^−1^): 3150, 1718 (CO), 1684 (CO), 1502, 1419, 1345, 1286, 1034, 761; ^1^H-NMR (DMSO-*d*_6_) 3.77 (s, 3H), 4.52 (d, *J* = 5.1 Hz, 2H), 5.20 (br t, *J* = 5.1 Hz, 1H), 7.04–7.12 (m, 1H), 7.25–7.33 (m, 2H), 7.36–7.45 (m, 2H), 7.45–7.58 (m, 4H), 7.68 (s, 1H); ^13^C-NMR (DMSO-*d*_6_) 54.9, 55.6, 79.3, 109.2, 118.4, 119.5, 124.7, 124.8, 128.7, 129.5, 130.3, 130.5, 134.6, 146.7, 155.1, 166.3, 188.9; HRMS (ESI+) calcd for C_19_H_17_N_4_O_4_ ([M + H]^+^) 365.1250, found 365.1267.

6-Methoxy-3-(4-phenyl-1H-1,2,3-triazol-1-yl)-3-propylquinoline-2,4(1H,3H)-dione (**3Ca**): Yellow solid; mp 288–315 °C (methanol-*N,N*-dimethylformamide); IR (cm^−1^): 3252, 1712 (CO), 1669 (CO), 1503, 1417, 1286, 1240, 1204, 763; ^1^H-NMR (DMSO-*d_6_*) 0.92 (t, *J* = 7.2 Hz, 3H), 1.21–1.50 (m, 2H), 2.50–2.65 (m, 2H), 3.81 (s, 3H), 7.20 (d, *J* = 8.9 Hz, 1H), 7.29 (d, *J* = 2.9 Hz, 1H), 7.31–7.43 (m, 2H), 7.47 (dd, *J* = 7.5, 7.5 Hz, 2H), 7.82–7.93 (m, 2H), 8.90 (s, 1H), 11.36 (br s, 1H); ^13^C-NMR (DMSO-*d_6_*) 13.7, 16.7, 38.2, 55.6, 74.6, 108.6, 118.4, 118.6, 122.3, 125.1, 125.6, 127.9, 128.9, 130.5, 135.7, 145.6, 155.2, 167.3, 190.5; HRMS (ESI+) calcd for C_21_H_21_N_4_O_3_ ([M + H]^+^): 377.1614, found 377.1617.

3-(4-(Hydroxymethyl)-1H-1,2,3-triazol-1-yl)-6-methoxy-3-propylquinoline-2,4(1H,3H)-dione (**3Cb**): Yellow solid; mp 189–191 °C (benzene-ethanol); IR (cm^−1^): 3083, 2912, 1715 (CO), 1686 (CO), 1509, 1422, 1335, 1182, 1172, 824; ^1^H-NMR (DMSO-*d_6_*) 0.89 (t, *J* = 7.2 Hz, 3H), 1.17–1.49 (m, 2H), 2.37–2.62 (m, 2H), 3.80 (s, 3H), 4.55 (br s, 2H), 5.25 (br s, 1H), 7.20 (d, *J* = 8.9 Hz, 1H), 7.26 (d, *J* = 3.0 Hz, 1H), 7.37 (dd, *J* = 8.9, 3.0 Hz, 1H), 8.24 (s, 1H), 9.50 (br s, 1H); ^13^C-NMR (DMSO-*d_6_*) 13.6, 16.7, 38.1, 54.9, 55.6, 74.4, 108.6, 118.5, 118.6, 123.6, 125.4, 135.7, 147.1, 155.1, 167.5, 190.5.; Anal. Calcd for C_16_H_18_N_4_O_4_ (330.34): C, 58.17; H, 5.49; N, 16.96. Found: C, 57.93; H, 5.45; N, 16.81.

*6-Chloro-7-methoxy-3-phenyl-3-(4-phenyl-1H-1,2,3-triazol-1-yl)quinoline-2,4(1H,3H)-dione* (**3Da**): Yellow solid; mp 300–304 °C dec. (DMF-ethanol); IR (cm^−1^): 3260, 1726 (CO), 1680 (CO), 1610, 1482, 1329, 1275, 1205, 763, 691; ^1^H-NMR (DMSO-*d_6_*) 3.93 (s, 3H), 6.78 (s, 1H), 7.30–7.36 (m, 1H), 7.42–7.48 (m, 4H), 7.52–7.56 (m, 3H), 7.80–7.88 (m, 2H), 8.41 (s, 1H), 11.68 (br s, 1H); ^13^C-NMR (DMSO-*d_6_*) 56.8, 79.2, 100.0, 112.4, 116.7, 123.2, 125.1, 127.8, 128.3, 128.7, 128.8, 129.5, 130.0, 130.3, 130.5, 141.7, 145.2, 160.6, 167.0, 186.4; HRMS (ESI+) calcd for C_24_H_18_ClN_4_O_3_ ([M + H]^+^): 445.1067, found 445.1047; Anal. Calcd for C_24_H_17_ClN_4_O_3_ (444.87): C, 64.80; H, 3.85; N, 12.59. Found: C, 64.55; H, 3.87; N, 12.35. 

*6-Chloro-3-(4-(hydroxymethyl)-1H-1,2,3-triazol-1-yl)-7-methoxy-3-phenylquinoline-2,4(1H,3H)-dione* (**3Db**): White solid; mp 279–283 °C dec. (ethanol); IR (cm^−1^): 2838, 1709, 1678, 1613, 1596, 1415, 1354, 1279, 1222, 1035; ^1^H-NMR (DMSO-*d*_6_) 3.92 (s, 3H), 4.52 (d, *J* = 5.0 Hz, 2H), 5.19 (br t, *J* = 5.0 Hz, 1H), 6.76 (s, 1H), 7.33–7.45 (m, 2H), 7.46–7.58 (m, 3H), 7.68 (s, 1H), 7.83 (s, 1H), 11.63 (br s, 1H); ^13^C-NMR (DMSO-*d*_6_) 54.9, 56.8, 78.9, 100.1, 112.5, 116.7, 124.6, 128.3, 128.6, 129.5, 130.3, 130.5, 141.8, 146.8, 160.6, 167.0, 186.5; Anal. Calcd for C_19_H_15_ClN_4_O_4_ (398.80): C, 57.22; H, 3.79; N, 14.05. Found: C, 57.05; H, 3.75; N, 13.92. 

*1-Benzyl-3-phenyl-3-(4-phenyl-1H-1,2,3-triazol-1-yl)quinoline-2,4(1H,3H)-dione* (**3Ea**): Yellow-brown solid; mp 105–113 °C (benzene-cyclohexane) IR (cm^−1^): 3061, 1714 (CO), 1679 (CO), 1600, 1468, 1453, 1373, 1312, 759, 695; ^1^H-NMR (DMSO-*d_6_*) 5.34 (d, *J* = 16.6 Hz, 1H), 5.48 (d, *J* = 16.6 Hz, 1H), 7.12–7.40 (m, 10H), 7.40–7.56 (m, 5H), 7.63 (dd, *J* = 7.7, 7.7 Hz, 1H), 7.84 (d, *J* = 7.5 Hz, 2H), 7.99 (d, *J* = 7.5 Hz, 1H), 8.56 (s, 1H); ^13^C-NMR (DMSO-*d_6_*) 26.3, 46.6, 80.2, 116.6, 120.8, 123.4, 124.0, 125.1, 126.7, 127.4, 127.9, 128.3, 128.6, 128.9, 129.5, 129.9, 130.5, 130.7, 135.7, 136.9, 140.8, 145.4, 166.9, 188.4; MS (EI) *m/z* (%): 471 ([M+1]^+^, 1), 470 ([M]^+^, 3), 326 (10), 206 (9), 116 (32), 92 (8), 91 (100), 77 (8); Anal. Calcd for C_30_H_22_N_4_O_2_ (470.52): C, 76.58; H, 4.71; N, 11.91; Found: C, 76.33; H, 4.73; N, 11.88. 

*1-Benzyl-3-(4-(hydroxymethyl)-1H-1,2,3-triazol-1-yl)-3-phenylquinoline-2,4(1H,3H)-dione* (**3Eb**). White solid; mp 184–187 °C (benzene-ethanol); IR (cm^−1^): 1721 (CO), 1691 (CO), 1601, 1468, 1453, 1373, 1311, 1027, 763, 693; ^1^H-NMR (DMSO-*d_6_*) 4.56 (d, *J* = 5.1 Hz, 2H), 5.23 (br t, *J* = 5.1 Hz, 1H), 5.31 (d, *J* = 16.7 Hz, 1H), 5.46 (d, J = 16.6 Hz, 1H), 7.15–7.39 (m, 9H), 7.41–7.55 (m, 3H), 7.61 (dd, *J* = 7.5, 7.5 Hz, 1H), 7.88 (s, 1H), 7.96 (d, *J* = 7.2 Hz, 1H); ^13^C-NMR (DMSO-*d_6_*) 46.7, 55.0, 80.0, 116.6, 121.0, 123.9, 124.9, 126.7, 127.4, 127.9, 128.6, 128.9, 129.5, 130.2, 130.6, 135.8, 136.8, 140.9, 147.0, 167.0, 188.5; MS (EI) *m/z* (%): 424 ([M]^+^, 2), 327 (16), 326 (16), 325 (9), 303 (7), 146 (7), 104 (10), 103 (7), 92 (9), 91 (100), 77 (11), 65 (10); Anal. Calcd for C_25_H_20_N_4_O_3_ (424.45): C, 70.74; H, 4.75; N, 13.20; Found: C, 70.49; H, 4.81; N, 12.91. 

### 3.7. One-Pot Synthesis of 3-Phenyl-3-(4-phenyl-1H-1,2,3-triazol-1-yl)quinoline-2,4(1H,3H)-dione (3Aa) Starting from 3-Bromo-3-phenylquinoline-2,4(1H,3H)-dione (6A)

A mixture of 3-bromo-3-phenylquinoline-2,4(*1H,3H*)-dione** (6A**, 316 mg, 1.00 mmol), pulverized NaN_3_ (130 mg, 2.00 mmol), phenylacetylene (**2a**, 103 mg, 1.01 mmol), CuSO_4_^.^5H_2_O (25 mg, 0.10 mmol), copper powder (280 mg, 4.41 mmol) and dimethyl sulfoxide (3.0 mL) was stirred in darkness for 20 h and then diluted with CH_2_Cl_2_ (80 mL). The reaction mixture was filtered and the filtrate was repeatedly extracted with saturated aqueous NH_4_Cl, until the aqueous phase remains colourless (totally 50 mL). The collected aqueous layer was back-extracted with CH_2_Cl_2_ (3 × 10 mL). The combined organic layers were dried over Na_2_SO_4_, filtered and evaporated *in vacuo*. Residual DMSO was removed by consecutive co-distillations *in vacuo* with toluene (2 × 15 mL) and then ethanol (2 × 15 mL). The product was suspended in cyclohexane (10 mL). The suspension was shortly boiled, cooled down to room temperature and the product was collected by filtration. Recrystallization from ethanol-acetic acid afforded pure **3Aa** (162 mg, 43%); mp 250–270 °C.

### 3.8. N-(3-(1-(1,2,3,4-Tetrahydro-2,4-dioxo-3-phenylquinolin-3-yl)-1H-1,2,3-triazol-4-yl)phenyl)acetamide ***(3Ae)***.

A suspension of 3-(4-(3-aminophenyl)-1*H*-1,2,3-triazol-1-yl)-3-phenylquinoline-2,4(1*H*,3*H*)-dione (**3Ac**, 99 mg, 0.25 mmol) in a mixture of ethyl acetate (0.5 mL) and pyridine (0.3 mL) was heated to the boiling point. Acetic anhydride (62 mmol, 6 mL) was added portion wise under shaking. The reaction mixture was cooled down to room temperature, left overnight and evaporated *in vacuo*. The residue was suspended in hot methanol (2 mL), cooled down to room temperature and product **3Ae** (72.3 mg, 65%) was collected by filtration: light brown powder; mp 307–314 °C (dec.); IR (cm^−1^): 3319, 1719, 1678, 1615, 1594, 1485, 1446, 1369, 784; ^1^H-NMR (DMSO-*d*_6_) d 2.06 (s, 3H), 7.11 (d, *J* = 8.1 Hz, 1H), 7.17 (dd, *J* = 7.5, 7.5 Hz, 1H), 7.35 (dd, *J* = 7.8, 7.8 Hz, 1H), 7.39–7.48 (m, 3H), 7.48–7.59 (m, 4H), 7.59–7.68 (m, 1H), 7.84 (dd, *J* = 7.7, 0.9 Hz, 1H), 8.10 (br s, 1H), 8.46 (s, 1H), 10.03 (br s, 1H), 11.62 (br s, 1H); ^13^C-NMR (DMSO-*d*_6_) d 24.0, 80.0, 115.5, 116.6, 118.4, 119.2, 119.8, 123.3, 127.4, 128.8, 129.2, 129.4, 129.9, 130.4, 130.8, 136.7, 139.8, 140.4, 145.2, 166.7, 168.3, 188.8; Anal. Calcd for C_25_H_19_N_5_O_3_ (437.45): C, 68.64; H, 4.38; N, 16.01. Found: C, 68.58; H, 4.39; N, 15.93.

## 4. Conclusions

In conclusion, 3-azidoquinoline-2,4(1*H*,3*H*)-diones readily undergo copper(I)-catalyzed [3 + 2] cycloaddition reaction with terminal alkynes to give the corresponding 1,4-disubstituted-1,2,3-triazoles. 
